# Molecular Modeling Identification of Key Secondary Metabolites from *Xylopia aethiopica* as Promising Therapeutics Targeting Essential Measles Viral Proteins

**DOI:** 10.1155/2023/1575358

**Published:** 2023-02-09

**Authors:** Jeremiah John Oloche, Bolaji Bosede Oluremi, Christiana Eleojo Aruwa, Saheed Sabiu

**Affiliations:** ^1^Department of Pharmacology and Therapeutics, College of Health Sciences, Benue State University of Makurdi, Makurdi, Nigeria; ^2^Department of Pharmacology and Therapeutics, College of Medicine, University of Ibadan, Oyo State, Ibadan, Nigeria; ^3^Department of Pharmaceutical Microbiology, University of Ibadan, Oyo State, Ibadan, Nigeria; ^4^Department of Biotechnology and Food Science, Faculty of Applied Sciences, Durban University of Technology, Durban, South Africa

## Abstract

This study computationally screened three key compounds (vanillin (VAN), oxophoebine (OPB), and dihydrochalcone (DHC)) derived from *Xylopia aethiopica* (Guinea pepper), a medicinal plant with known antiviral activity, against key druggable measles virus (MV) proteins (fusion protein (FUP), haemagglutinin protein (HMG), and phosphoprotein (PSP)). Each molecular species was subjected to a 100 ns molecular dynamics (MD) simulation following docking, and a range of postdynamic parameters including free binding energy and pharmacokinetic properties were determined. The docking scores of the resulting OPB-FUP (−5.4 kcal/mol), OPB-HMG (−8.1 kcal/mol), and OPB-PSP (−8.0 kcal/mol) complexes were consistent with their respective binding energy values (−25.37, −28.74, and −40.68 kcal/mol), and higher than that of the reference standard, ribavirin (RBV) in each case. Furthermore, all the investigated compounds were thermodynamically compact and stable, especially HMG of MV, and this observation could be attributed to the resulting intermolecular interactions in each system. Overall, OPB may possess inhibitory properties against MV glycoproteins (FUP and HMG) and PSP that play important roles in the replication of MV and measles pathogenesis. While OPB could serve as a scaffold for the development of novel MV fusion and entry inhibitors, further *in vitro* and *in vivo* evaluation is highly recommended.

## 1. Introduction

Measles is a communicable disease caused by a member of the genus *Morbillivirus*, and species *Measles morbillivirus*, also referred to as the measles virus (MV) [1, 2]. The measles virus infects the central nervous system leading to serious neurological disorders. Despite the availability of clinically effective live attenuated vaccines currently in use, the disease is one the major cause of morbidity and mortality largely among unvaccinated children. The disease accounted for approximately 140,000 global deaths in children below 5 years in 2018, with most of the reported cases occurring in Africa and the Eastern Mediterranean regions [3]. Recently, over a thousand children were reportedly infected in a measles outbreak in North-East Nigeria [4].

The measles virus is an enveloped antigenically monotypic negative-sensesingle-stranded RNA virus [5]. The measles virus core consists of an RNA genome covered by a nucleocapsid protein that is surrounded by an envelope composed of glycoproteins such as haemagglutinin (*H*) and fusion (*F*) proteins [6].

**Figure 1:**
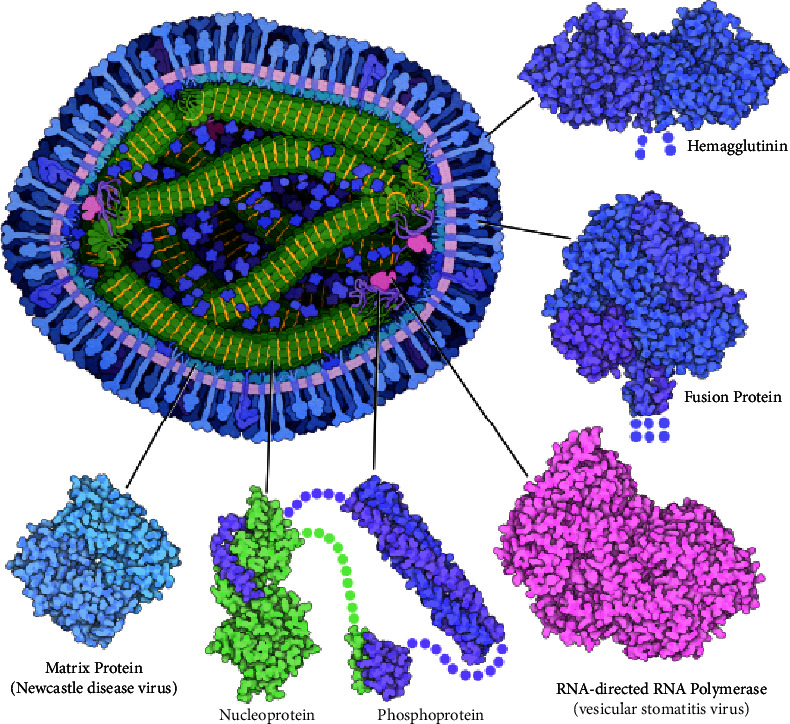
A cross-section of measles viral particle showing the significant proteins in the pathogenesis of measles virus infection (adapted from https://pdb101.rcsb.org/motm/231).

The pathogenesis of measles virus infection is highly coordinated and involves an initial binding of H protein (Figure 1) to host cell surface receptor targets, namely, the signaling lymphocyte activation molecule (CD150 or SLAM), CD 46, and the adherent junction protein PVRL4 (nectin-4) receptors [7]. The measles virus H protein head domain distinctively binds to a *β*-sheet of the membrane distal ectodomain of SLAM by its *β*-propeller fold and then triggers a conformational change on the F protein and extension into the target membrane [8, 9]. The *H*protein-host surface receptor (SLAM) interaction provides an excellent antimeasles virus drug target. The measles virus fusion (*F*) protein (Figure 1) plays an all-important function in the fusion of the virus and entry into the host cell. Membrane fusion machinery generated by the conformational change of *F* protein on the host cell leads to viral-cell membrane merging and cell-cell viral spread [9]. A second *F* protein conformational change results in the formation of fusion pores and subsequent entry of the genetic material (nucleocapsid) into the cytoplasm of the infected cell [5, 9].

While measles vaccines have been clinically administered over the years, the two recommended doses do not confer immunity in up to 10% of individuals due to inadequate development of antibodies [10]. Global eradication of the disease would require a 95% herd immunity that is achievable by the synergistic use of antimeasles virus drugs coupled with vaccination [5]. Unfortunately, there are presently no specific drugs approved for use in acute complications or persistent measles virus infections [11].

**Figure 2:**
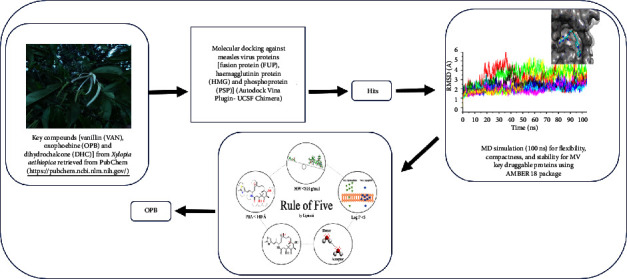
Workflow of the strategy adopted.

The resurgence of measles virus (MV) infection, a common cause of childhood morbidity and mortality especially in developing countries despite the availability of clinically effective measles vaccines [3, 4], requires a concerted effort in the discovery of antimeasles therapeutics to reduce its global human health impact. Previous research studies by Krumm and collaborators [12] reported a potential oral measles virus polymerase inhibitor. However, there is a dearth of information on antimeasles virus drugs. Therefore, this research study was designed to screen compounds isolated from *Xylopia aethiopica* that have been reported to demonstrate notable antimeasles virus activity *in vitro* [13] by employing molecular dynamics (MD) simulation techniques against essential druggable viral proteins of MV. Furthermore, in silico pharmacokinetic evaluation of the most promising compound was also undertaken and the entire protocols adopted in this study are presented in Figure 2.

## 2. Methodology

### 2.1. Acquisition and Preparation of Target Proteins and Compounds

The X-ray crystal structures of measles virus fusion protein (FUP), haemagglutination (HMG) protein, and phosphoprotein (PSP) with PDB codes 5YZC, 2ZB5, and 5E4V, respectively, were sourced from the RSCB Protein Data Bank (https://www.rcsb.org/). Water molecules and residue connectivity were removed from the protein structures, and the missing side chains were added to the structures using the UCSF Chimera software V1.14 [14, 15]. The 2D structures of phytocompounds, oxophoebine (OPB), dihydrochalcone (DHC), and vanillin (VAN) and the ribavirin (RBV) standard were retrieved from PubChem (https://pubchem.ncbi.nlm.nih.gov/). Optimization of the 3-D structures of the compounds that were used for the molecular docking was achieved by adding Gasteiger charges and nonpolar hydrogen atoms using Avogadro software [16, 17].

### 2.2. Molecular Docking

The prepared compounds (OPB, DHC, VAN, and RBV) were docked into the binding sites of measles virus fusion protein (FUP), haemagglutination protein (HMG), and phosphoprotein (PSP), using the Autodock Vina Plugin on Chimera. The grid box was defined with a spacing of 1 Å and appropriate sizes pointing in *x*, *y,* and *z* directions in each case. The docked complexes that showed the finest pose based on the docking scores were further subjected to molecular dynamics (MD) simulation.

### 2.3. Molecular Dynamics Simulation

The molecular dynamics (MD) simulation was performed by the method described by Idowu et al. [18]. The MD simulation was performed utilizing the GPU version provided with the ., in which the FF18SB variant of the AMBER force field was used to describe the systems [19]. The restrained electrostatic potential and the general amber force field procedures were used to generate atomic partial charges for the compounds, and the addition of hydrogen atoms, sodium, and chloride countered ions for neutralization of all systems in an ANTECHAMBER of the leap module of AMBER 18 [20]. The amino acid residues were numbered 1–81 for FUP, 1–330 for HMG, and 1–315 for PSP. The system was then suspended implicitly within an orthorhombic box of TIP3P water molecules such that all atoms were within 8 Å of any box edge.

First, 2000 steps of minimization were performed for the solutes in the initial 1,000 steps following the method of steepest descent, and a subsequent 1000 steps of conjugate gradients with an applied restraint potential of 500 kcal/mol. A further 1000 steps of full minimizations were performed by employing the conjugate gradient algorithm with no restraint. Stepwise heating from 0 to 300 K MD simulation was carried out for 50 ps in a manner that a fixed number of atoms and at a fixed volume were maintained and the systems equilibrated at 500 ps. A potential harmonic restraint of 10 kcal/mol and a collision frequency of 1.0 ps were imposed on the systems containing the solutes. In order to mimic an isobaric-isothermal ensemble (NPT), the operating temperature was fixed at 300 K using the Langevin thermostat, and the pressure was kept constant at 1 bar using the Berendsen barostat [21, 22], with randomized seeding, and a pressure-coupling constant of 2 ps [23]. The SHAKE algorithm was employed to constrict the bonds of hydrogen atoms during the MD simulations and was conducted for a period of 100 ns at a step size of 2 fs using the SPFP precision model [24, 25].

### 2.4. Postdynamic Analysis

Root mean square deviation (RMSD), root means square fluctuation (RMSF), solvent accessible surface area (SASA), and radius of gyration (RoG) were analyzed using the CPPTRAJ module employed in the AMBER 18 suite, and the raw data plots were subsequently generated by employing the Origin data analysis software [26].

### 2.5. Binding Free Energy Calculations

The free binding energy for each molecular species was calculated using the molecular mechanics with the generalized born surface area method (MM/GBSA) [27], and the binding affinity of the systems was compared. The mean binding free energy estimate was determined for over 100000 snapshots extracted from the 100 ns trajectory. Mathematically, the computation of the free binding energy (∆*G*_bind_) by employing this method is represented in the following equations:

(1) $$\begin{array}{c} ∆G_{bind}=G_{complex}\;–\;G_{receptor}\;–\;G_{ligand}\text{,}\\ ∆G_{bind}=E_{gas}+G_{sol}\;–\;TS\text{,}\\ E_{gas}=E_{\mathrm{int}}+E_{vdw}+E_{ele}\text{,}\\ G_{sol}=G_{GB}+G_{SA}\text{,}\\ G_{SA}=\gamma SASA\text{,} \end{array}$$

where *E*_gas_ = gas-phase energy, *E*_int_ = internal energy, *E*_elec_ = Coulomb energy, *E*_vdw_ = van der Waals energies, *G*_sol_ = solvation free energy, GSA = nonpolar solvation energy, SASA = solvent-accessible surface area, GGB = polar solvation, *S* = total entropy of the solute, and *T* = temperature.

### 2.6. Analysis of Pharmacokinetic Properties of Test Compounds

The pharmacokinetic properties of the potential lead compound(s) were predicted using SwissADME online platform (http://swissadme.ch/index.php) as described by Daina et al. [28].

### 2.7. Data Analysis

Descriptive statistics and the OriginPro software (V9.10) were used to present ∆*G*_bind_ and all raw data plots, respectively [26].

## 3. Results

The molecular docking analysis for all the research compounds against the possible MV targets is presented in Table 1. The compounds VAN, OPB, and DHC had docking scores of −3.7, −5.4, and −4.5 kcal/mol, respectively, on FUP relative to −4.6 kcal/mol observed for the standard antiviral agent, RBV. The docking score of OPB on HMG was −8.1 kcal/mol and it was significantly higher when compared to the docking scores of VAN, −5.5 kcal/mol, and DHC, −6.6 kcal/mol. However, the docking score of OPB on HMG was comparable to the docking score of RBV at −7.0 kcal/mol. Findings from this study showed that the docking scores of OPB and RBV against PSP were −5.9 and −4.4 kcal/mol, respectively (Table 1).

Table 1 further shows the energy component profiles, including the binding free energy (∆*G*_bind_) values obtained through the 100 ns exposure period of the investigated systems. The binding affinities of OPB on measles virus FUP, HMG, and PSP were −25.37, −28.74, and −40.68 kcal/mol, respectively, and were significantly higher compared to the ∆*G*_bind_ of VAN, DHC, and RBV on the same targets.

**Figure 3:**
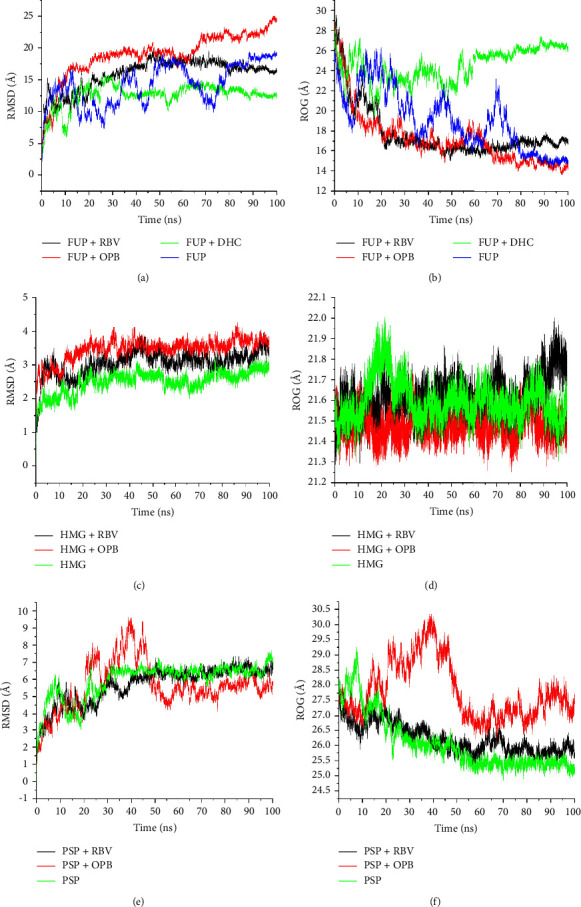
Comparative plots of the FUP, HMG, and PSP complexes with OPB, DHC, and RBV presented as RMSD (a), (c), and (e) and RoG (b), (d), and (f). FUP = fusion protein, PSP = phosphoprotein, and HMG = haemagglutinin protein.

The data on the predicted structural and conformational changes with the potential to modify the functioning of the investigated targets presented as RMSD, RoG, and RMSF of alpha carbon (*Cα*) atoms are shown in Table 2, while the plots are presented in Figures 3(a)–3(f).

Figure 3(a) shows the RMSD value of the OPB-FUP complex (18.96 Å) that was higher than the RMSD values of the RBV-FUP complex (15.92 Å) and unbound FUP (14.30 Å). Observed RMSD values of the molecules OPB (3.45 Å) and RBV (3.05 Å) exhibited relatively similar deviations when in an HMG system. However, the unbound system produced an RMSD value of 2.50 Å (Table 2 and Figure 3(c)). The RMSD values for the unbound PSP and the complexes formed were higher than the values derived from the unbound HMG and its complexes (Table 2 and Figure 3(e)). The RoG plot of FUP-OPB and FUP-RBV systems produced average RoG values of 17.19 and 17.71 Å, respectively. These values were lower than 24.48 and 19.11 Å produced by DHC and the unbound FUP, respectively (Table 2 and Figure 3(b)). However, average RoG values of HMG and PSP systems with the experimental drug molecules and the respective unbound proteins were similar (Table 2 and Figures 3(d) and 3(f)).

**Figure 4:**
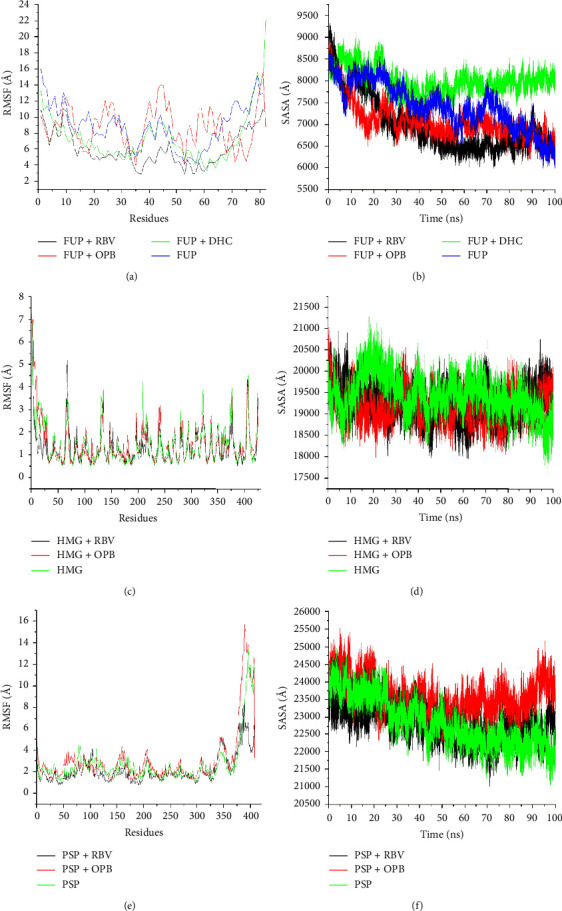
Comparative plots of FUP, HMG, and PSP with molecules OPB, DHC, and RBV presented as RMSF (a), (c), and (e) and SASA (b), (d), and (f). FUP = fusion protein, HMG = haemagglutinin protein, and PSP = phosphoprotein.

The root mean square fluctuation (RMSF) values of alpha carbon atoms after molecular dynamics simulation of the target proteins-ligands interactions are presented in Figure 4. Oxophoebine gave the highest observed RMSF value of 8.78 Å and was comparable with 8.92 Å for the unbound FUP system. The standard antiviral, RBV and DHC recorded RMSF values of 6.04 Å and 7.43 Å, respectively, and these were lower than that of the unbound FUP (Table 2 and Figure 4(a)). The RMSF values of OPB (1.43 Å) and RBV (1.32 Å) were similar to 1.44 Å recorded for the unbound HMG system. In PSP systems, the RSMF values of OPB, RBV, and the unbound PSP system were calculated to be 3.08, 2.17, and 2.68 Å, respectively (Table 2 and Figures 4(b) and 4(c)). The SASA investigations of the FUP complex systems showed respective average values of 7080.96 Å and 8007.82 Å for OPB and DHC against RBVs average of 6963.91 Å; while the average values were closely comparable for unbound HMG protein and the complex system it formed with OPB and RBV standards (Table 2 and Figures 4(d) and 4(e)). A similar observation was drawn for the unbound PSP protein and PSP-OPB complex, but the PSP-OPB complex had the highest average SASA value at 23578.47 Å (Table 2 and Figure 4(f)).

**Figure 5:**
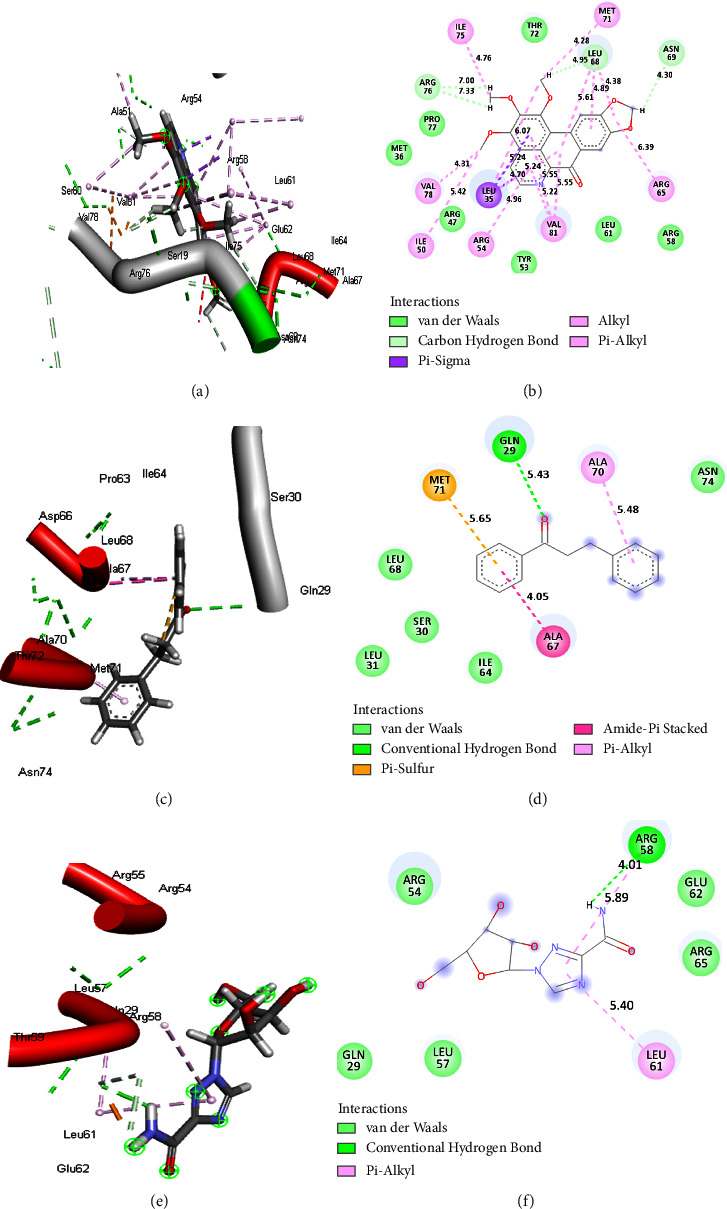
The 3D (a), (c), and (e) and 2D (b), (d), and (f) interaction plots of FUP-OPB, FUP-DHC, and FUP-RBV, respectively, with 18, 9, and 7 bonds. FUP = measles virus fusion protein, OPB = oxophoebine, DHC = dihydrochalcone, and RBV = ribavirin.

**Figure 6:**
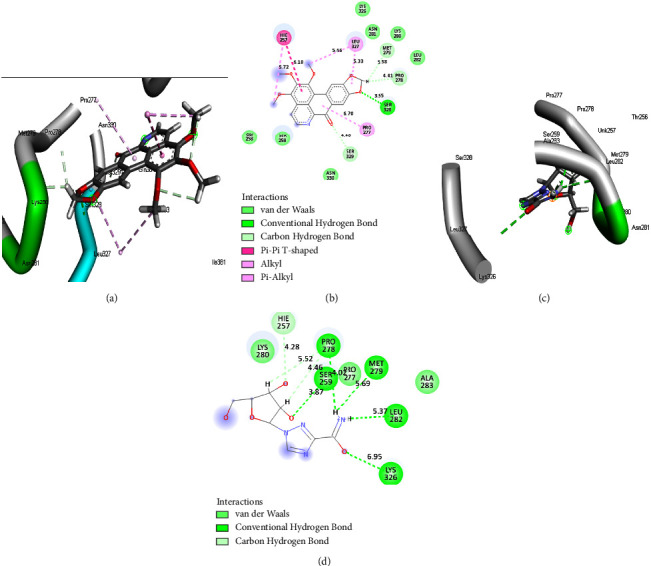
The 3D (a) and (c) and 2D (b) and (d) interaction plots of HMG-OPB and HMG-RBV systems with 14 and 9 bonds, respectively. HMG = measles virus haemagglutinin protein, OPB = oxophoebine, and RBV = ribavirin.

**Figure 7:**
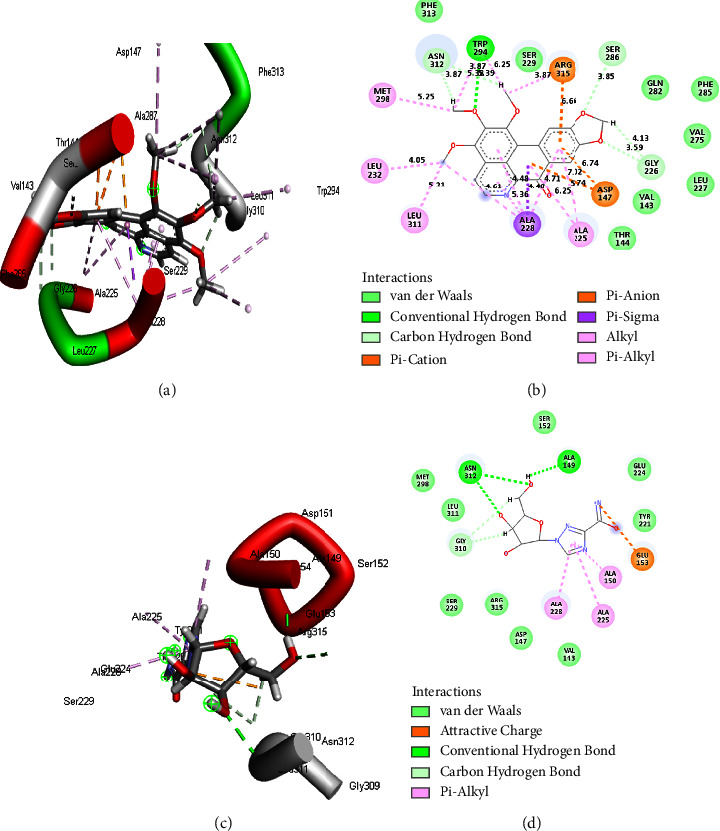
The 3D (a) and (c) and 2D (b) and (d) interaction plots of PSP-OPB and PSP-RBV systems with 19 and 16 bonds, respectively. PSP = measles virus phosphoprotein, OPB = oxophoebine, and RBV = ribavirin.

The results from post-MD simulation interactions in FUP bound systems with OPB showed 18 interactions, compared to 9 and 7 interactions for DHC and RBV, respectively (Figures 5(a)–5(f)). Oxophoebine interacted with 14 amino acid residues of the HMG protein forming van der Waal's bonds, as well as conventional H (Ser328), 2 alkyl (Pro277 and Leu327), 3 C-H (Met279, Pro278, and Ser329), and 1 pi-pi*T*-shaped (His257) interactions (Figures 6(a) and 6(b)). On the other hand, a total of 9 interactions were observed for the HMG-RBV system including 3 C-H (Lys280, Ala283, and Pro277), van der Waal's, and conventional hydrogen interactions (Figures 6(c) and 6(d)). The PSP-bound systems with OPB and RBV showed 19 and 16 total interactions, respectively (Figures 7(a)–7(d)). The PSP-OPB interactions were conventional H (Trp294), 3 C-H (Ser286, Asn312, and Gly226), 4 alkyl, 1 pi-Cation (Arg315), 1 pi-anion (Asp147), 1 pi-sigma (Ala228), and van der Waal's bonds (Figures 7(a) and 7(b)), while the PSP-RBV system had conventional H, 1 C-H (Gly310), 3 pi-alkyl, 1 attractive charge (Glu153), and van der Waal's bonds interactions (Figures 7(c) and 7(d)).

The pharmacokinetic and physicochemical properties of the promising compound, oxophoebine, and ribavirin are presented in Table 3. The molecular weights and log *P* of oxophoebine (365.34 g/mol and 2.97) and ribavirin (244.20 g/mol and 0.13) were below 500 g/mol and 5, respectively. Oxophoebine was predicted to have high gastrointestinal absorption, while ribavirin was predicted to have low gastrointestinal absorption. The experimental compound (oxophoebine) and the standard antiviral drug share the same bioavailability score of 0.55 and the same number of hydrogen acceptors which is 7, but below 10.

## 4. Discussion

In this study, three compounds isolated from *Xylopia aethiopica* were computationally evaluated against key structural proteins of MV and the higher docking scores observed with OPB and DHC relative to the low score of VAN could be an indication of better fitness and orientation of OPB and DHC at the binding sites of FUP, HMG, and PSP. The scoring provides the necessary information to predict the binding affinity of the prospective drug to the target [29]. Binding energy calculations and MD simulation indicated that OPB and DHC showed better poses or best fit on the MV target proteins. As such, these were preferentially selected and subsequently subjected to MDS analysis. The MM/GBSA technique estimates the total binding free energy (∆*G*_bind_) of an inhibitor that could cause structural and conformational changes that may alter the biological activities of the target protein [18, 30, 31]. The higher negative binding affinity for the MV proteins, FUP, HMG, and PSP observed with OPB in this study may indicate better interactions that could lead to alterations in the normal functioning of these proteins. Although, the binding affinity observed for DHC on FUP was significantly lower compared to OPB on FUP, and it was rather comparable to ribavirin (RBV), the standard antiviral agent. This observation suggests that DHC may not significantly alter the normal biological activities of the MV fusion protein.

The RMSD measures the degree of convergence, stability, or deviations produced by a protein in a simulation system [32]. In this study, though the lower mean RMSD of the FUP-DHC system relative to the unbound FUP could imply that DHC stabilized FUP than OPB and RBV, but this was, however, not consistent with the thermodynamic binding energy value and may represent a pseudostability of the complex. The trend for the simulated OPB-HMG was not different from that observed with the FUP system. In contrast to this observation, the marginally lower mean RMSD value of the PSP-OPB complex than that of the apo PSP compared with RBV could be suggestive of OPB's potential as a prospective lead compound, and perhaps a novel inhibitor of MV PSP. Interestingly, this result agrees with the thermodynamic profiles where the lowest binding free energy correlates with greater compactness and structural stability of the complex. The compound, OPB lowered the mean RoG value of FUP resulting in more structural compactness and stability compared to the unbound FUP which further suggests that OPB is a potential inhibitor of FUP. However, in the case of HMG, the RoG value of the protein-bound molecules were like those of the unbound protein and OPB seemed not to have changed the compactness of the target protein indicating that HMG may not be an important target for the inhibitory activity of OPB. The flexibility and structural compactness of the bound target proteins is a function of the radius of gyration (RoG), and binding of the drug molecule to the protein may lead to a change in biological function. A low RoG value for a drug molecule is an indication of a more stable system relative to the unbound protein [31].

The RMSF value is used to determine the fluctuations at the active site of a protein when bound to a drug molecule, and higher or lower *α*-carbon fluctuations imply more or less flexible movements, respectively [33]. This justifies the determination of RMSF value in this research study. In this study, similar fluctuation patterns were seen in the residues, 0–10, 75–200, and 100–350 in all the FUP, HMG, and PSP systems, respectively. The estimated RMSF values of OPB-FUP or OPB-HMG systems were not different from those of the unbound proteins which indicates that the compound did not exact any reasonable dynamic alterations at the active sites of the target proteins residues and is predictive of the inhibitory activity of OPB. The SASA is useful to determine the surface area of the drug-protein complex accessibility to solvents, and the impact on the drug molecule on SASA [34]. When solvent accessibility surface area values are high, it indicates reduced structural/system stability and increased surface area, and vice versa [35]. The respective lower SASA values of the OPB-HMG and OPB-FUP systems relative to the standard indicated reduced protein residue-solvent molecule interactions and targets' surface area. These results suggested increased systems stability and emphasized the compounds' potential as treatment or drug options against such targets. However, OPB caused the most significant structural entropy with all target proteins studied compared to other test compounds. This observation is in agreement with its lower free binding energy values against all targets compared to the standard.

Compounds with inhibitory activity usually bind to amino acids at the active sites of a protein to inactivate it. However, binding is dependent on thermodynamic parameters linked to protein interaction types, protein compactness, and the stability and flexibility of the amino acid residues [36, 37]. The number, nature, and length of bonds of interactions of the test drug candidates and standard antiviral drugs at the active sites of FUP, HMG, and PSP proteins varied, and it impacted the reported binding free energy values. Among observed bond, interactions were the C-H bonds, conventional hydrogen bonds, pi-piT-shaped, van der Waals, amide pi-stacked, pi-cation, pi-anion, pi-sigma, pi-pi-stacked, and attractive charges. The OPB-bound systems generally had the highest number of interactions with all protein targets, which was in direct agreement with the high ∆*G*_bind_ scores for OPB-bound systems compared to DHC and RBV-bound systems. The number of hydrogen bonds was also highest in OPB compound-protein systems with exception of HMG-RBV systems. Hydrogen bonds have unusually strong interactions between molecules and constitute one of the most essential bonds in drug discovery [38]. Nonetheless, the HMG-RBV system interactions had higher average bond lengths (5.02 Å) compared to the HMG-OPB (4.48 Å) complex system and accounted for the low free binding energy of the former. Bond length (BL) is important in determining the affinity/tightness of atoms, and shorter BLs contribute to stronger intramolecular or intermolecular pull, greater atomic hold, and affinity [39, 40]. Again, among the test compounds simulated, only oxophoebine (OPB) interacted with a hydrogen bond linked to an essential amino acid residue, Trp294, at the active site of PSP (Figure 6). This gives an insight into the compound's affinity for the PSP active site and could be responsible for the observed higher binding free energy.

In this study, OPB showed a potential inhibitory effect on the biological activity of the HMG and fusion proteins of the MV. The effect of OPB as a potential MV fusion inhibitor could be similar to its blockage of the human immunodeficiency virus (HIV)-1 which envelops glycopeptides fusion with the cell membrane of host CD4 cells [41], as well as the anti-MV effects of the small compound, AS-48 via binding to hydrophobic pockets in the region of measles virus fusion protein [42]. The PSP, much like the nucleoprotein, also binds to the RNA polymerase during viral replication [43, 44]. Interference with the process of PSP and RNP complex formation leads to altered transcription and efficiency in viral particle assembly, viral replication inhibition, or death. The inhibition of PSP by OPB may be closely linked to this mechanism of action, that is, via PSP-RNP complex formation. This effect, like that of small molecules previously identified and characterized by Krumm et al. [12] could ultimately result in the inhibition of viral replication and death of MV. Measles virus HMG and fusion proteins mediate the binding of the viral envelope to host cell surface proteins and the process of virus-cell fusion, respectively. Inhibition of the HMG protein-host would interfere with the interaction between MV surface receptor, signaling lymphocyte activation molecule (SLAM) that might result in the inhibition of the required initial conformational changes of the fusion protein, a rate-limiting step in the pathogenesis of MV infection. In addition, compounds with an antifusion activity that interfere with conformational changes of FUP would exhibit potent measles virus fusion inhibition [6, 9]. The measles virus HMG and fusion proteins are therefore promising targets for the discovery and development of MV fusion inhibitors [11].

Pharmacokinetics study showed log *P* values of the OPB compound and the standard drug, ribavirin to be less than 5, suggesting good lipid membrane permeability and absorption, and the potential of OPB formulation for oral administration. In-silico pharmacokinetics values give an overview of *in vivo* drug interactions and decrease the chances of failure during drug development [45]. Lipinski's rule of five (Ro5) gives an indication of the physicochemical properties, drug-likeness, and safety properties of prospective bioactive compounds intended for oral administration [46]. The Ro5 states that compounds that have log *P* values >5 may poorly permeate the lipid membrane and not pass through the gut walls [47, 48]. A high rate of absorption in the gastrointestinal tract (GIT) is a major consideration during oral drugs design [49]. Again, both OPB and RBV showed a good bioavailability score of 0.55. A drug's bioavailability score (the proportion of a drug dose that remains unchanged and reaches the systemic circulation) is also key in drug dose calculations [50]. A high bioavailability score indicates a high drug concentration needed to elicit maximum therapeutic effects at the site of action. Still, bioactive molecules with low oral bioavailability could be formulated for administration via nonoral routes [50]. The compound OPB was also predicted to permeate the blood-brain barrier (BBB), but not the standard drug. It hence possesses an added advantage if used in the management of MV-induced encephalomyelitis. The BBB prevents the passage of toxic compounds of molecular weight greater than 400 g/mol [51]. Our results suggest that both OPB and RBV molecules are good drug candidates for oral administration against MV. Lastly, this study's findings also support the previous *in vitro* antimeasles virus report on *Xylopia aethiopica* [13], with OPB as the most promising compound responsible for the elicited activity.

## 5. Conclusion

The glycoproteins (haemagglutinin and fusion proteins) and phosphoprotein play integral functions in the replication of the measles virus and pathogenesis of measles, thus, finding potent and clinically useful inhibitors for these proteins is undoubtedly important in the treatment of measles disease. The drug candidate, oxophoebine (OPB), that showed similar or better physicochemical and pharmacokinetic properties compared to the FDA-approved antiviral drug, ribavirin might possess multiple-target inhibitory activities against the measles virus. Hence, this compound could serve as a scaffold for the development of inhibitors of MV fusion, haemagglutinin proteins, and phosphoproteins.

## Figures and Tables

**Table 1:** Docking score and thermodynamic binding free energy of the compounds with MV druggable targets.

Target/compound	Docking score (kcal/mol)	Energy components (kcal/mol)				
		Δ*E*_vdW_	Δ*E*_elec_	∆*G*_gas_	∆*G*_solv_	∆*G*_bind_
*FUP*						
VAN	−3.7	—	—	—	—	—
RBV	−4.6	−20.42 ± 4.1	−14.53 ± 7.7	−34.95 ± 9.6	17.72 ± 6.3	−17.23 ± 4.5
OPB	−5.4	−33.30 ± 6.0	−8.09 ± 3.9	−41.40 ± 8.4	16.03 ± 4.4	−25.37 ± 5.1
DHC	−4.5	−16.51 ± 11.2	−2.42 ± 3.5	−18.93 ± 13.0	5.79 ± 4.7	−13.14 ± 9.1

*HMG*						
VAN	−5.5	—	—	—	—	—
RBV	−7.0	−25.16 ± 4.6	−31.50 ± 13.2	−56.66 ± 14.6	37.63 ± 10.2	−19.03 ± 6.7
OPB	−8.1	−41.85 ± 4.4	−16.84 ± 5.3	−58.70 ± 8.0	29.95 ± 4.2	−28.74 ± 5.1
DHC	−6.6	—	—	—	—	—

*PSP*						
VAN	−5.0	—	—	—	—	—
RBV	−4.4	−28.77 ± 4.9	−18.76 ± 15.2	−47.53 ± 15.1	28.09 ± 10.9	−19.43 ± 6.4
OPB	−8.0	−51.46 ± 2.9	−17.43 ± 3.9	−68.89 ± 5.4	28.20 ± 3.3	−40.68 ± 3.7

VAN = vanillin; OPB = oxophoebine; DHC = dihydrochalcone; RBV = ribavirin; FUP = fusion protein; HMG = haemagglutinin protein; PSP = phosphoprotein; Δ*E*_elec_ = electrostatic energy; Δ*E*_vdW_ = van der Waals energy; ∆*G*_bind_ = total binding free energy; ∆*G*_solv_ = solvation free energy; Δ*E*_gas_ = gas phase free energy. Data presented are ± standard deviation of average values, — = not determined.

**Table 2:** Postmolecular dynamics simulation analysis of interactions of compounds of *Xylopia aethiopica* with measles virus druggable targets.

Target/compound	Average RMSD (Å)	Average RMSF (Å)	Average RoG (Å)	Average SASA (Å)
FUP	14.30 ± 3.19	8.92 ± 2.70	19.11 ± 2.96	7433.19 ± 531.60
FUP-RBV	15.92 ± 2.43	6.04 ± 2.20	17.71 ± 2.57	6963.91 ± 650.40
FUP-OPB	18.96 ± 3.55	8.78 ± 2.57	17.19 ± 2.64	7080.96 ± 435.40
FUP-DHC	12.58 ± 1.85	7.43 ± 3.09	24.48 ± 1.61	8007.82 ± 291.40

HMG	2.50 ± 0.32	1.44 ± 0.10	21.59 ± 0.095	19429.50 ± 399.60
HMG-RBV	3.05 ± 0.36	1.32 ± 0.11	21.62 ± 0.087	19288.04 ± 440.85
HMG-OPB	3.45 ± 0.33	1.43 ± 0.14	21.48 ± 0.061	19217.43 ± 348.90

PSP	5.92 ± 1.02	2.68 ± 2.03	26.07 ± 0.89	22837.87 ± 682.54
PSP-RBV	5.66 ± 1.07	2.17 ± 1.29	26.23 ± 0.89	22795.77 ± 682.54
PSP-OPB	5.62 ± 1.42	3.08 ± 2.64	27.79 ± 0.94	23578.47 ± 505.89

FUP = fusion protein; HMG = haemagglutination protein; PSP = phosphoprotein; RBV = ribavirin; OPB = oxophoebine; DHC = dihydrochalcone; RMSD = root mean square deviation; RMSF = root means square fluctuation; RoG = radius of gyration; SASA = solvent accessible surface area. Data presented are ± standard deviation of average values.

**Table 3:** Comparison of predicted ADMET properties of oxophoebine and ribavirin.

Property	Molecule (ligand)	
	Oxophoebine (OPB)	Ribavirin (RBV)
Mol. formula	C_20_H_15_NO_6_	C_8_H_12_N_4_O_5_
Mol. weight (g/mol)	365.34	244.20
Lipophilicity (iLog *P*)	2.97	0.13
Water solubility	Moderate	Low
GIT absorption	High	Low
BBB permeability	Yes	No
Hydrogen bond acceptors	7	7
Hydrogen bond donors	0	4
Bioavailability score	0.55	0.55
Drug likeness (Lipinski)	Yes	Yes

Mol. = molecular; Log *P* = partition coefficient; GIT = gastrointestinal tract; BBB = blood-brain barrier.

## Data Availability

The data used to support the findings of this study are included within the article.
